# Endoscopic ultrasound-guided recanalization of pancreaticojejunostomy anastomotic stricture using a forward-viewing echoendoscope

**DOI:** 10.1055/a-2791-4846

**Published:** 2026-02-17

**Authors:** Shota Iwata, Takuji Iwashita, Takuya Koizumi, Yosuke Ohashi, Akinori Maruta, Shinya Uemura, Masahito Shimizu

**Affiliations:** 1 476117 First Department of Internal Medicine, Gifu University Hospital, Gifu, Japan; 2 13051 Department of Gastroenterology, Shiga University of Medical Science, Shiga, Japan

Endoscopic ultrasound-guided transmural pancreatic duct drainage (EUS-PDD) has become an important alternative when transpapillary or transanastomotic pancreatic duct drainage is technically challenging 1 2 . Recently, forward-viewing echoendoscope (FV-EUS)-guided drainage through the anastomotic site has been reported as a novel EUS-guided drainage technique; however, this approach has rarely been evaluated.

**Fig. 1:**
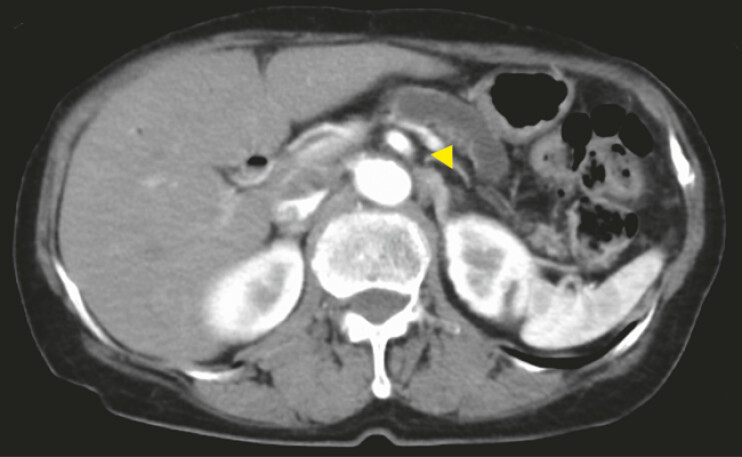
Computed tomography shows the dilation of the main pancreatic duct (yellow arrowhead).

**Fig. 2:**
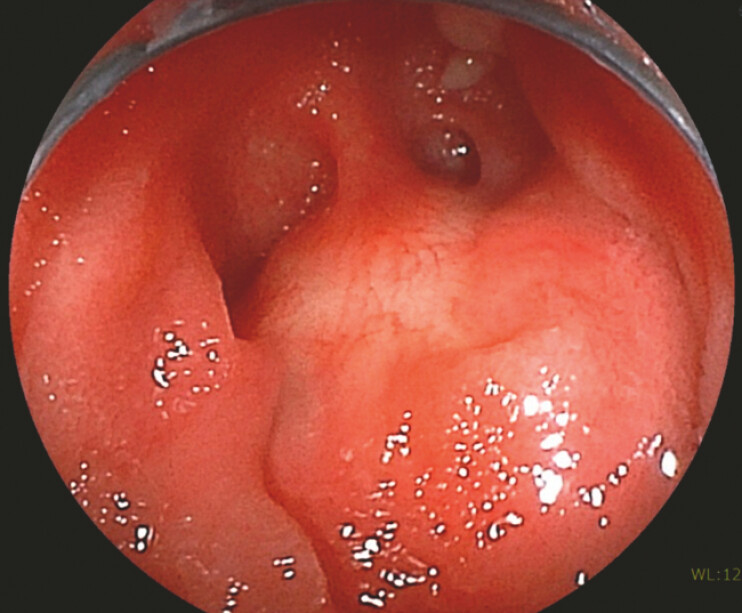
The opening of the pancreatic duct could not be identified at the pancreaticojejunostomy site.

**Fig. 3:**
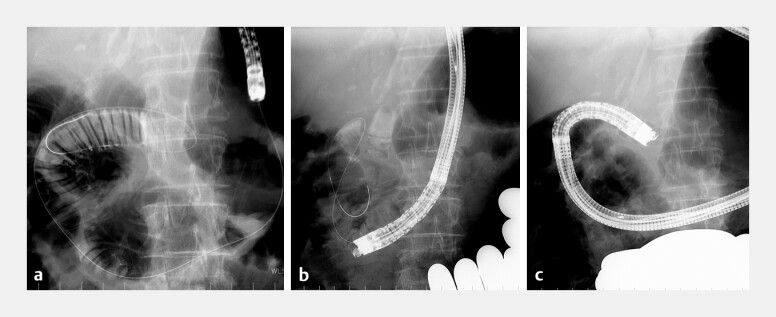
**a** A guidewire was left in place at the anastomosis, and the balloon endoscope was withdrawn. **b** External manual abdominal compression enabled the advancement of the echoendoscope along the guidewire. **c** The echoendoscope was successfully inserted at the anastomotic site.

**Fig. 4:**
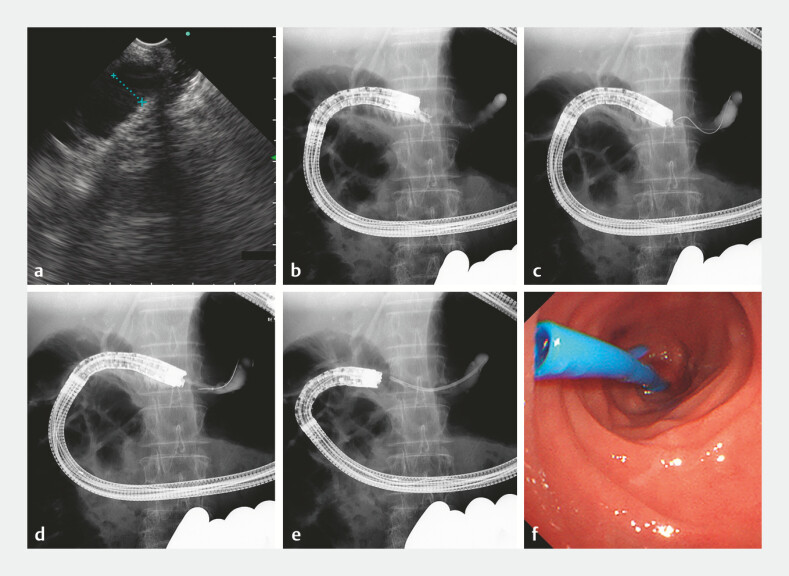
**a** EUS showed the dilated pancreatic duct through the anastomosis. **b** The pancreatic duct was punctured through the anastomosis. **c** A 0.025-inch guidewire was inserted into the pancreatic duct. **d** The tract was dilated using a 4-mm balloon. **e** A 7-Fr, 7-cm plastic stent was deployed across the anastomotic stricture. **f** An endoscopic view of the deployed stent. EUS, endoscopic ultrasound

A 69-year-old woman presented with epigastric discomfort 4 years after undergoing subtotal stomach-preserving pancreaticoduodenectomy for a pancreatic head neuroendocrine tumor (NET G1). Computed tomography demonstrated dilation of the main pancreatic duct (15 mm) indicating a pancreaticojejunostomy anastomotic stricture (PJAS; Fig. 1 ). Double-balloon endoscopy-assisted endoscopic retrograde cholangiopancreatography was attempted; however, the opening of the pancreatic duct could not be identified although the scope reached the anastomosis ( Fig. 2 ). Therefore, EUS-guided recanalization using FV-EUS (TGF-UC260J, Olympus, Tokyo, Japan) was planned. With the aid of a guidewire placed near the anastomosis ( Fig. 3 **a** ) and external manual compression, FV-EUS was successfully advanced to the anastomotic site ( Fig. 3 **b, c** ). The dilated main pancreatic duct (9 mm) was punctured at the anastomotic site using a 19-gauge needle (Sonotip, Medi-Globe, Achenmühle, Germany), followed by contrast injection to confirm proper puncture ( Fig. 4 **a, b** ). A 0.025-inch guidewire was advanced into the pancreatic duct ( Fig. 4 **c** ). The tract was dilated using a 4-mm REN balloon (KANEKA MEDIX, Osaka, Japan; Fig. 4 **d** ), and finally, a 7-Fr, 7-cm plastic stent was deployed ( Fig. 4 **e, f** , Video 1 ). The patient’s symptoms improved after the procedure, without adverse events.

EUS-guided recanalization of the pancreaticojejunostomy anastomotic stricture using a forward-viewing echoendoscope, including follow-up imaging. EUS, endoscopic ultrasound.Video 1

This case indicated that EUS-guided recanalization of the PJAS using FV-EUS was feasible and safe. Compared with conventional transmural EUS-PDD, EUS-guided recanalization may offer improved safety because the access route is through the PJA rather than through the abdominal cavity and pancreatic parenchyma, if FV-EUS can reach the anastomotic site 3 4 5 . Further studies are required to clarify the safety, feasibility, and long-term outcomes of this technique.

Endoscopy_UCTN_Code_TTT_1AS_2AI
